# Significance of Serum Angiopoietin-2 in Patients with Hemorrhage in Adult-Onset Moyamoya Disease

**DOI:** 10.1155/2020/8209313

**Published:** 2020-08-04

**Authors:** Jianbo Yu, Kaiyuan Huang, Jianwei Pan, Jian Shen, Renya Zhan

**Affiliations:** Department of Neurosurgery, First Affiliated Hospital, School of Medicine, Zhejiang University, Hangzhou Zhejiang Province, China

## Abstract

**Background:**

Moyamoya disease (MMD) is a progressive occlusive cerebrovascular disease that is characterized by abnormal angiogenesis at the base of the brain. This pathological abnormal angiogenesis is susceptible to disturbances, including spontaneous hemorrhage and vasogenic edema. However, the underlying mechanisms of pathological angiogenesis and occurrence of hemorrhage are unclear. Angiopoietins play a fundamental role in the pathophysiology of central nervous system disorders in angiogenesis. This study was aimed at examining whether angiopoietins are associated with formation of abnormal collateral vessels and the occurrence of hemorrhage in adult-onset moyamoya disease (HMMD).

**Methods:**

A total of 27 consecutive adult patients with HMMD were enrolled from June 2011 to May 2017. Serum levels of angiopoietin-1 (Ang-1) and angiopoietin-2 (Ang-2) were examined by enzyme-linked immunosorbent assay. Patients with HMMD were compared with those with spontaneous hemorrhage (controls) and nonhemorrhagic-onset MMD (NHMMD).

**Results:**

Serum Ang-2 levels were significantly higher in patients with adult HMMD than in those with spontaneous hemorrhage and NHMMD. The ROC curve identified that a baseline serum Ang-2 level > 1230 ng/ml may be associated with adult HMMD with 88.39% sensitivity and 70.37% specificity (area under the curve (AUC), 0.89; 95% CI, 0.808-0.973; *P* < 0.001). Moreover, serum Ang-2 levels were significantly elevated in stages II, III, and IV. In subgroup analysis of a high and low degree of moyamoya vessels, serum Ang-2 levels were significantly higher in the high moyamoya vessel group than in the low moyamoya vessel group. Serum Ang-2 levels were also significantly higher in the low moyamoya vessel group compared with the control group. Serum Ang-1 levels were not significantly different among the groups.

**Conclusion:**

Increased serum Ang-2 levels may contribute to pathological abnormal angiogenesis and/or to the instability of vascular structure and function, thus causing brain hemorrhage in adult HMMD.

## 1. Introduction

Moyamoya disease (MMD) is a rare cerebrovascular disease. MMD is characterized by progressive stenosis or occlusion of the terminal portion of internal carotid arteries and the formation of a vascular network at the base of the brain [1–3]. Formation of abnormal collateral vessel shows two different clinical patterns of pathological changes including cerebral ischemic events due to stenoocclusive changes at the circle of Willis arteries (particularly in children) and/or the occurrence of cerebral hemorrhage (particularly in adults) [4]. An abnormal vascular network is assumed to be an increased angiogenic response to progressive ischemia [5]. Pathological angiogenesis increases microvascular density and the microvascular diameter [6]. This process involves compensatory recruitment for blood supply. However, pathological angiogenesis can be vulnerable to disruption, including vasogenic edema and spontaneous hemorrhage [7]. Although there have been many extensive studies on pathological angiogenesis [7], the underlying mechanisms and the occurrence of spontaneous hemorrhage are still undetermined.

Angiopoietins, which are a recently discovered distinct family of angiogenic proteins, have recently been shown to play fundamental roles in pathophysiological angiogenesis of central nervous system disorders [8–11]. Specifically, angiopoietin-1 (Ang-1) directly antagonizes vascular hyperpermeability by promoting interendothelial cell–cell stability and regulating antiadhesive and anti-inflammatory effects on endothelial cells [12–14]. Angiopoietin-2 (Ang-2) is an inflammatory and apoptotic mediator in endothelial cells and promotes expression of adhesion molecules, facilitating leukocyte migration, which induces vascular permeability [9, 15]. Recently, many studies have shown that, unlike tumor angiogenesis, vascular endothelial growth factor (VEGF) does not play a major role in observed angiogenesis in patients with MMD [5, 16], Additionally, IL-8, PDGF, EGF, and TGF-*β* are not mechanisms of angiogenesis in MMD [5, 17, 18]. However, expression of angiopoietins in adult hemorrhage-onset MMD (HMMD) has not been examined previously. Therefore, this study was aimed at examining serum levels of Ang-1 and Ang-2 in patients with adult HMMD to identify the roles and mechanism of abnormal angiogenesis and the occurrence of hemorrhage.

## 2. Materials and Methods

### 2.1. Ethics Statement

This study was approved by the ethics committee of the First Affiliated Hospital, School of Medicine, Zhejiang University, and each participant signed written informed consent before enrollment.

### 2.2. Patients and Examinations

A total of 27 consecutive adult patients with HMMD were admitted to the neurocritical care unit between June 2011 and May 2017. Diagnostic cerebral angiography or computed tomography angiography was performed during the first 12 h after admission. Patients with the following features were excluded from this study: (1) a history of infective, inflammatory, neoplastic, or hematological diseases, organ infarct, or trauma and (2) complications of cerebral arteriovenous malformation, cavernous hemangioma, or aneurysms. Moreover, 12 consecutive adult patients with nonhemorrhagic-onset MMD (NHMMD group) were also included during the same period. To avoid the biological effect of cerebrovascular hemorrhage, 27 patients with spontaneous intracerebral hemorrhage were also recruited with the hemorrhage location and volume matched with the HMMD group (control group) during the same period for controls. The hemorrhagic etiology of cerebral arteriovenous malformation, cavernous hemangioma, and aneurysm and the condition of (1) were excluded.

Angiographic examinations were performed by two neuroradiologists (JS and JWP) or neuroradiology fellows or residents under the supervision of the neuroradiologists. Angiographic analysis of the moyamoya stage in intracranial arteries was performed using the Suzuki classification [3]. Each neuroradiologist reviewed angiographic imaging unless a question or doubt existed. If a question was raised, more neuroradiologists made the decision after discussion. According to the degree of richness of moyamoya vessels by Suzuki classification [3], patients with MMD were divided into two subgroups of high moyamoya vessels and low moyamoya vessels. High moyamoya vessels were defined as Suzuki stages II, III, and IV, and low moyamoya vessels were defined as Suzuki stages I, V, and VI.

### 2.3. Sample Collection and Measurement

Venous blood samples were drawn from the patients with HMMD on admission and prior to surgery and were collected within 3 days from the bleeding onset using Sarstedt Monovette serum tubes. Serum of blood samples was obtained by centrifugation at 3000 × g for 15 min within 2 h after at least 30 min of clotting blood time and stored at −80°C until use. Ang-1 and Ang-2 levels were measured in serum samples using an enzyme-linked immunosorbent assay (R&D Systems, Minneapolis, MN, USA) according to the manufacturer's instructions. Ang-1 and Ang-2 levels were quantified twice in each patient, and the mean value of each marker was used as the result.

### 2.4. Statistical Analysis

Data are shown as mean ± standard deviation. Multiple group differences were analyzed using ANOVA. If the overall ANOVA revealed significant differences, the comparisons between groups were performed by Bonferroni test. Receiver operating characteristic (ROC) curves and area under the ROC curves were configured to establish the cutoff point of Ang-2 with the optimal sensitivity and specificity (maximization the sum of sensitivity and specificity) for discriminating between the HMMD group and control group. Ninety-five percent confidence intervals (95% CIs) were estimated according to the binomial distribution. Two-tailed *P* values of <0.05 were considered statistically significant. Statistical analysis was performed using SPSS (San Diego, CA).

## 3. Results

### 3.1. Patients' Characteristics

In adult HMMD, the patients' age ranged from 24 to 59 years, and there were 13 men and 14 women. There were three patients in Suzuki classification I, six in Suzuki classification II, seven in Suzuki classification III, six in Suzuki classification IV, three in Suzuki classification V, and two in Suzuki classification VI. Twelve patients had hypertension, and 10 were smokers. The characteristics of adult HMMD are shown in Table 1. In NHMMD, the patients' age ranged from 23 to 67 years, and there were five men and seven women. Four patients had hypertension, and seven were smokers. There were three patients in Suzuki classification I, one in Suzuki classification II, two in Suzuki classification III, two in Suzuki classification IV, two in Suzuki classification V, and two in Suzuki classification VI. Two patients had no symptoms, six had transient ischemic attack, and four had cerebral infarction. In the spontaneous hemorrhage control group, 12 patients had basal ganglia hemorrhage, 11 had intraventricular hemorrhage, three had lobar hemorrhage, and one had SAH. The patients' age ranged from 16-62 years, and there were 11 men and 16 women. Fourteen patients had hypertension, and nine were smokers. Baseline characteristics of the groups are shown in Tables 2 and 3.

### 3.2. Serum Ang-1 and Ang-2 Levels in HMMD

**Figure 1:**
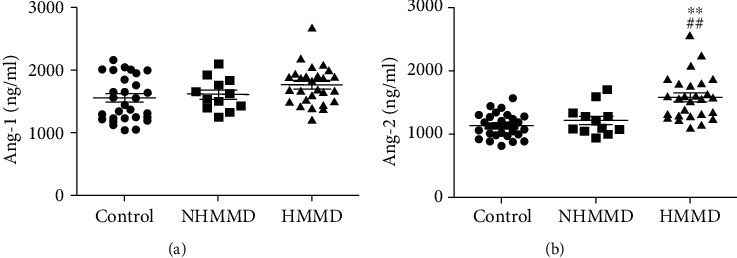
Serum levels of Ang-1 and Ang-2 in patients with adult HMMD, NHMMD, and controls. (a) There was no difference in serum Ang-1 levels among the groups. (b) Serum Ang-2 levels were significantly higher in the adult HMMD group than in the control and NHMMD groups (both *P* < 0.01). ^∗∗^*P* < 0.01*vs.* the control group, ^##^*P* < 0.01*vs.* the NHMMD group.

**Figure 2:**
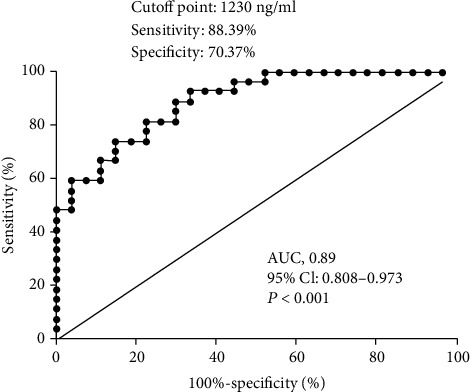
The ROC curve analysis of plasma Ang-2 concentration for predicting the occurrence of HMMD. The ROC curve identified that that serum Ang-2 levels > 1230 ng/ml may predict the occurrence of adult HMMD with 88.39% sensitivity and 70.37% specificity (area under curve (AUC), 0.89; 95% CI, 0.808-0.973; *P* < 0.001).

**Figure 3:**
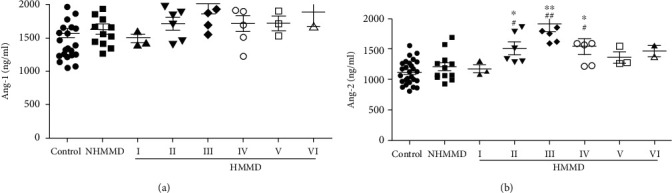
Serum levels of Ang-1 and Ang-2 in patients with Suzuki stage classification, those with NHMMD, and controls. (a) Serum Ang-1 levels were not significantly different among the stages of MMD. (b) Serum Ang-2 levels were significantly elevated at stages II (*P* < 0.05), III (*P* < 0.01), and IV (*P* < 0.05) compared with NHMMD and controls. ^∗^*P* < 0.05*vs.* the control group,^∗∗^*P* < 0.01*vs.* the control group, ^#^*P* < 0.05*vs.* the NHMMD group, and ^##^*P* < 0.01*vs.* the NHMMD group.

**Figure 4:**
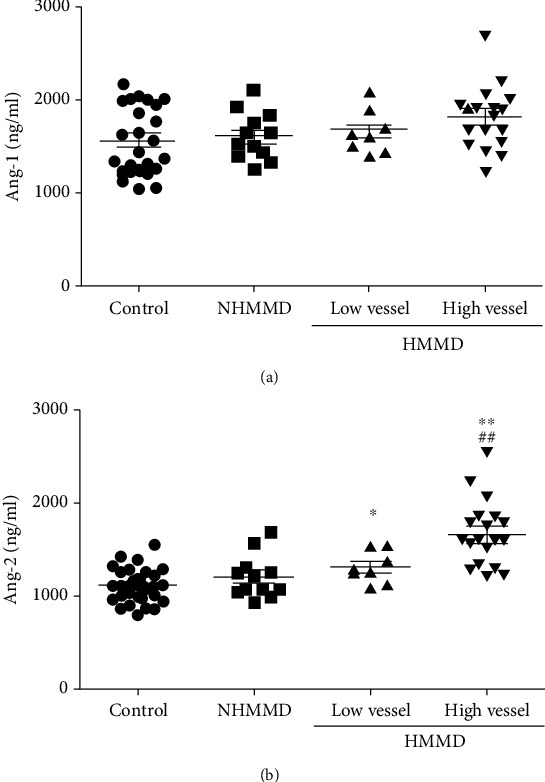
Serum levels of Ang-1 and Ang-2 in patients in the high moyamoya vessel group, low moyamoya vessel group, NHMMD group, and control group. (a) There was no difference in serum Ang-1 levels among the groups. (b) Serum Ang-2 levels were significantly higher in the high moyamoya vessel group than in the low moyamoya vessel group (*P* < 0.01). Ang-2 levels were also significantly higher in the low moyamoya vessel group than in the control group (*P* < 0.05). ^∗^*P* < 0.05*vs.* the control group, ^∗∗^*P* < 0.01*vs.* the control group, and ^##^*P* < 0.01*vs.* the low vessel group.

Serum Ang-1 levels were not significantly different among the groups (Figure 1(a)). In contrast, serum Ang-2 levels were significantly higher in patients with adult HMMD group than in the control and NHMMD groups (both P <0.01) (Figure 1(b)). Moreover, a ROC curve identified that a baseline serum Ang-2 level > 1230 ng/ml were associated with adult HMMD with 88.39% sensitivity and 70.37% specificity (area under curve (AUC), 0.89; 95% CI, 0.808-0.973; *P* < 0.001) (Figure 2). Ang-1 and Ang-2 serum levels were also measured at Suzuki stages I–VI for comparison among the groups. Serum Ang-1 levels showed no significant association with the Suzuki stage of MMD (Figure 3(a)). In contrast, serum Ang-2 levels were significantly elevated at stages II (*P* < 0.05), III (*P* < 0.01), and IV (*P* < 0.05) compared with the control group (Figure 3(b)). In subgroup analysis, serum Ang-2 levels were significantly higher in the high moyamoya vessel group than in the low moyamoya vessel group (*P* < 0.01). Furthermore, serum Ang-2 levels were significantly higher in the low moyamoya vessel group compared with the control group (*P* < 0.05) (Figure 4(b)). There was no significant difference in serum Ang-1 levels among the groups. However, there was a trend towards higher Ang-1 serum levels in patients in the adult HMMD group than in the control and NHMMD group (Figure 4(a)).

## 4. Discussion

Adult-onset MMD, which is most common in Asian populations, is mainly characterized by development of one or more spontaneous cerebral hemorrhages. Up to 66% of patients with adult-onset MMD present with hemorrhage, the majority of which occurs in the ventricular system, thalamus, and the basal ganglia [19, 20]. Such hemorrhages are usually repetitive in nature, with an annual rate of recurrent bleeding of 7% [20, 21]. A pathological characteristic of hemorrhage in MMD is formation of basal, fragile angiogenesis, which involves thin and dilated vessels often associated with significant vessel fibrosis and marked attenuation of the media.

Microaneurysms, angionecrosis, and increased hemodynamic shear stress are considered etiologies for cerebral hemorrhage, which often occur in adult-onset MMD [7, 22]. Many extensive studies have suggested that ischemia, hypoxia, inflammation, and infection are involved in the history of moyamoya vessels in MMD [5, 23–25]. However, the pathophysiological mechanisms involved in moyamoya vessels still remain unknown. Angiopoietins, which are prominent regulators of vascular development, are promising biomarkers that play fundamental pathophysiological roles in the central nervous system, such as traumatic brain injury, brain tumors, cerebrovascular disease, and inflammation [8, 10, 11, 15]. Ang-1 and Ang-2 might be the most representative members of the angiopoietin family, acting as an agonist of the Tie-2 receptor, whereas Ang-2 is an antagonist [26]. These agonist and antagonist activities offer important insights into the pathophysiological mechanisms involved in endothelial cell integrity, blood–brain barrier (BBB) permeability, and vascular responsiveness in different disease states. Therefore, the primary hypothesis is that angiopoietins are altered and associated with the degree of moyamoya vessels and the occurrence of hemorrhage with adult HMMD.

Ang-2 is only weakly expressed in endothelial cells under physiological conditions. However, Ang-2 expression is dramatically upregulated under pathological conditions. Several studies have helped to establish the role of serum Ang-2 levels as a marker of secondary damage with systemic disease [8, 27]. Previous studies have also indicated that imbalanced serum Ang-2 levels have prognostic implications in patients [27, 28]. Elevated serum Ang-2 levels are associated with the acute physiology and chronic health evaluation score, the organ failure index, and injury severity score. High serum Ang-2 levels have adverse outcomes in a wide range of human diseases, such as cancer, neuroendocrine tumors, infectious diseases, inflammatory, rheumatoid arthritis, and malaria [29, 30]. Serum Ang-2 levels are also associated with an increased risk of recurrent stroke in patients with cerebral infarction [31]. The present study showed that serum Ang-2 levels were significantly higher in patients with adult HMMD, and the ROC curve also identified that serum Ang-2 levels > 1230 ng/ml may be associated with the occurrence of adult HMMD with 88.39% sensitivity and 70.37% specificity. Although some previous studies also demonstrated that Ang-2 expression was significantly overexpressed in the M3 segment of MCA from patients with MMD [32, 33], the upregulation of Ang-2 in MCA did not influence the concentration of Ang-2 in the circulation of MMD patients [32]. However, these results are not contradictory to our research, because the conclusion is based on the comparison with atherosclerotic cerebrovascular disease. But in our study, a significant increase was only observed in patients with adult HMMD than NHMMD and control group, and serum Ang-2 levels were also not significantly increased in the NHMMD group compared with control. Moreover, the damage of BBB may lead to the increase in plasma Ang-2 levels in adult HMMD.

In our study, Ang-2 levels were significantly elevated at Suzuki stages II–IV compared with the other stages. According to the Suzuki classification, moyamoya vessels gradually increase from stages II–IV and then gradually disappear from stages V–VI. So, we made an attempt to define Suzuki stages II, III, and IV as high moyamoya vessel and Suzuki stages I, V, and VI as low moyamoya vessel for subgroup analysis. We also found that Ang-2 levels were significantly higher in the low moyamoya vessel group compared with the control group. Moreover, we also found that most adult patients with HMMD (70%) presented with a high degree of moyamoya vessels, which is consistent with previous reports [4, 19]. These results indicate that Ang-2 may promote proliferation of moyamoya vessels with adult HMMD. Therefore, Ang-2 might promote formation of fragile, basal, moyamoya collateral vessels and, at least in part, facilitate disruption of abnormal moyamoya vessels that are vulnerable to intracerebral hemorrhage.

The underlying hemorrhagic mechanism of high serum Ang-2 levels in adult HMMD is unclear. This finding could be explained by several reasons. First, Ang-2 is generally considered to be a proinflammatory cytokine in destruction of blood vessels. The Ang-2-deficient mouse cannot elicit an inflammatory response in *Staphylococcus aureus*-induced or thioglycollate-induced peritonitis [9]. In a classical inflammatory state, such as sepsis and rheumatoid arthritis, serum Ang-2 levels are significantly increased and associated with C-reactive protein, which is a sensitive marker of inflammation [28, 34]. Therefore, Ang-2 levels are considered to indicate an association between vascular proliferation and inflammation. Second, Ang-2 is also considered to be an active modulator of BBB breakdown. In a previous study, injection of Ang-2 in the normal rat cortex caused a significant breakdown of the BBB with vascular endothelial cell apoptosis in the pia and peripheral lesions [35]. Moreover, Ang-2 increases the expression level of matrix metalloproteases, which are able to digest the endothelial basal lamina. This indicates that Ang-2 plays a major role in maintaining BBB impermeability through regulation of tight junctions, resulting in opening of the BBB [36]. Third, generally, Ang-2 and VEGF act together to promote neovascularization via mediating vasodilation and remodeling of the basal lamina and proliferation and migration of endothelial cells [8]. However, strong angiogenic factors, such as VEGF, are not involved in angiogenesis in MMD [5, 16]. Therefore, Ang-2 may separately promote endothelial cell death and vascular regression in angiogenesis when VEGF is not present or prevented in MMD [37]. Finally, a previous study showed that overexpression of Ang-2 resulted in aberrant and intact angiogenesis with poor smooth muscle cell coverage [37]. This previous finding indicates that the intrinsic vulnerability to form a cerebral aneurysm is the main cause of hemorrhage in MMD. Overall, Ang-2 induces vascular destabilization and leakage in the course of angiogenesis.

Ang-1 is another important regulator of angiogenesis and vascular homeostasis. Ang-1 stabilizes the endothelial cell-to-cell adherens and tight junctions by increasing expression of endothelial junctional complexes and occludens [13, 14]. The present study showed that serum Ang-1 levels were not significantly different among the groups. Therefore, Ang-1 may not play an important role in pathophysiological mechanisms of fragile, basal, moyamoya collateral vessels and occurrence of spontaneous hemorrhage with adult HMMD.

There are some limitations to our research. First, the number of patients who were enrolled in the study was relatively small. Moreover, temporary serum levels of angiopoietins were not measured with development of MMD. Additionally, serum levels may be not able to completely show histological changes. These issues may lead to bias in understanding the role of angiopoietins in the history of pathophysiological mechanisms of adult HMMD. A strength of our study is providing novel information on the significance of angiopoietins in pathophysiological mechanisms in adult HMMD.

In conclusion, our study shows, for the first time, that Ang-2 may play an important role in pathophysiological mechanisms of augmented angiogenesis in adult HMMD. Ang-2 may also be a prognostic factor of brain hemorrhage in patients with adult MMD.

## Figures and Tables

**Table 1:** Summary of adult hemorrhage-onset MMD recruited in our research.

Case	Age	Sex	Presentation	Angiographic findings	Cardiovascular history	Suzuki stage
1	45	F	R BGH	Bilateral ICA intracranial occlusion	Hypertension	III
2	49	M	L BGH	L MCA occlusion R ICA intracranial occlusion	Smoke	IV
3	29	M	SAH	Bilateral ICA intracranial stenosis	None	I
4	42	M	IVH	L ICA intracranial stenosis R ICA intracranial occlusion	Hypertension, smoke	IV
5	51	M	L BGH+IVH	L MCA intracranial stenosis	Smoke	I
6	38	F	R FLH	R MCA intracranial stenosis	None	IV
7	53	F	IVH	R ICA intracranial stenosis	None	III
8	24	F	L TLH	Bilateral ICA intracranial occlusion	None	V
9	59	F	R BGH	L ICA intracranial stenosis	Hypertension, smoke	I
10	36	M	IVH	Bilateral ICA intracranial occlusion	None	II
11	42	M	L BGH	Bilateral ICA intracranial occlusion	None	III
12	51	M	IVH	Bilateral ICA intracranial occlusion	Hypertension, smoke	VI
13	46	F	L BGH+IVH	L ICA intracranial stenosis R ICA intracranial occlusion	None	IV
14	48	F	IVH	R ICA intracranial occlusion	Smoke	II
15	53	F	R BGH+IVH	Bilateral ICA intracranial stenosis	Hypertension	IV
16	34	F	R TLH	Bilateral ICA intracranial occlusion	None	III
17	56	F	R BGH	R ICA intracranial stenosis	Hypertension	II
18	46	M	IVH	Bilateral ICA intracranial occlusion	Smoke	VI
19	38	M	L BGH+IVH	Bilateral ICA intracranial stenosis	None	III
20	31	M	IVH	Bilateral ICA intracranial occlusion	Hypertension, smoke	V
21	36	F	L BGH	L ICA intracranial stenosis R ICA intracranial occlusion	Hypertension, smoke	II
22	49	M	IVH	R ICA intracranial occlusion	Hypertension	II
23	45	F	R BGH+IVH	Bilateral ICA intracranial occlusion	Hypertension	V
24	29	F	IVH	L ICA intracranial occlusion R ICA intracranial stenosis	None	IV
25	34	M	IVH	L ICA intracranial stenosis R ICA intracranial occlusion	Hypertension	II
26	44	F	IVH	Bilateral ICA intracranial occlusion	Smoke	III
27	57	M	L BGH	L ICA intracranial occlusion L MCA occlusion	Hypertension	III

BGH: basal ganglia hemorrhage; F: female; FLH: frontal lobe hemorrhage; ICA: internal carotid artery; IVH: intraventricular hemorrhage; L: left; M: male; R: right; SAH: subarachnoid hemorrhage; TLH: temporal lobe hemorrhage.

**Table 2:** Baseline characteristics of adult HMMD, NHMMD, and control group.

Characteristics	Control	NHMMD	HMMD	*P*
Age (years)	43.70 ± 11.76	42.92 ± 13.24	43.15 ± 9.37	0.59
Gender (M/F)	11/16	5/7	13/14	0.96
Cardiovascular history				
Hypertension (yes/no)	14/13	4/8	12/15	0.27
Smoke (yes/no)	9/18	7/5	10/17	0.77
Suzuki stage (I/II/III/IV/V/VI)	Non	3/1/2/2/2/2	3/6/7/6/3/2	0.66^&^
Hemorrhage volume (ml)	20.16 ± 5.62	None	16.98 ± 4.80^∗^	0.13

^∗^ *n* = 26 (one patient with SAH was excluded for uncertainty of the volume).Values are expressed as means ± standard deviation. ^&^Pearson's *χ*^2^ test.

**Table 3:** Baseline characteristics of low vessel, high vessel HMMD, NHMMD, and control group.

Characteristics	Control	NHMMD	Low vessel HMMD	High vessel HMMD	*P*
Age (years)	43.70 ± 11.76	42.92 ± 13.24	43.11 ± 9.02	43.7 ± 11.76	0.99
Gender (M/F)	11/16	5/7	5/3	8/11	0.66
Cardiovascular history					
Hypertension (yes/no)	14/13	4/8	4/4	8/11	0.90
Smoke (yes/no)	9/18	7/5	5/3	5/14	0.22
Hemorrhage volume (ml)	20.16 ± 5.62	Non	16.09 ± 5.87	17.38 ± 4.85^∗^	0.21

∗*n* = 18 (one patient with SAH was excluded for uncertainty of the volume).Values are expressed as means ± standard deviation.

## Data Availability

The data used to support the findings of this study are available from the corresponding author upon request.
